# Is Avoiding an Aversive Outcome Rewarding? Neural Substrates of Avoidance Learning in the Human Brain

**DOI:** 10.1371/journal.pbio.0040233

**Published:** 2006-07-04

**Authors:** Hackjin Kim, Shinsuke Shimojo, John P O'Doherty

**Affiliations:** **1**Division of Humanities and Social Sciences, California Institute of Technology, Pasadena, California, United States of America; **2**Division of Biology, California Institute of Technology, Pasadena, California, United States of America; Johns Hopkins UniversityUnited States of America

## Abstract

Avoidance learning poses a challenge for reinforcement-based theories of instrumental conditioning, because once an aversive outcome is successfully avoided an individual may no longer experience extrinsic reinforcement for their behavior. One possible account for this is to propose that avoiding an aversive outcome is in itself a reward, and thus avoidance behavior is positively reinforced on each trial when the aversive outcome is successfully avoided. In the present study we aimed to test this possibility by determining whether avoidance of an aversive outcome recruits the same neural circuitry as that elicited by a reward itself. We scanned 16 human participants with functional MRI while they performed an instrumental choice task, in which on each trial they chose from one of two actions in order to either win money or else avoid losing money. Neural activity in a region previously implicated in encoding stimulus reward value, the medial orbitofrontal cortex, was found to increase, not only following receipt of reward, but also following successful avoidance of an aversive outcome. This neural signal may itself act as an intrinsic reward, thereby serving to reinforce actions during instrumental avoidance.

## Abstract 2

Functional imaging suggests a similar role for the human medial orbitofrontal cortex in processing the receipt of a reward and the successful avoidance of an aversive outcome.

## Introduction

In instrumental conditioning an animal or human learns to increase the frequency of a response that leads to a rewarding outcome or else leads to avoidance of an aversive outcome. Psychological accounts of instrumental conditioning have, since the law of effect, held that receipt of a rewarding outcome in a given context serves to strengthen or *reinforce* associations between that context and the response performed, thereby ensuring that such a response is more likely to be selected in the future [ 1, 2]. Such a notion also forms the basis of modern computational theories of reinforcement learning, in which actions leading to greater predicted reward are reinforced via an afferent reward prediction error signal, encoding discrepancies between actual and expected reward at the time of outcome [ 3– 9]. Reinforcement-based theories are supported by a wide range of behavioral and neural data garnered from studies of instrumental reward learning in both animals and humans [ 10– 14].

Unlike reward learning, avoidance learning is a form of instrumental conditioning not so easily accounted for by standard theories of reinforcement. The problem is that once an aversive outcome has been successfully avoided, the individual no longer experiences explicit reinforcement for their behavior, and thus, behavior appears to be maintained even in the absence of reinforcement [ 15]. Yet according to reinforcement theory, such behavior should rapidly extinguish. The fact that responding appears to be maintained even in extinction runs counter to the basic tenets of reinforcement theory.

Various explanations have been advanced to account for this apparent paradox, including two factor theories advocating interactions between instrumental and Pavlovian learning processes [ 16] or theories invoking cognitive expectancies [ 17]. Each of these theories has received some experimental support [ 18, 19]. However, perhaps the most parsimonious theoretical account is to propose that in avoidance learning, successfully avoiding an aversive outcome itself, acts as a reward. Thus, avoidance behavior is positively reinforced on each trial when the aversive outcome is avoided, just as receipt of reward reinforces behavior during reward conditioning [ 20, 21]. In this sense, avoidance of an aversive outcome could be considered to be an “intrinsic reward,” with the same positive reinforcing properties as a real “extrinsic” reward. This account is grounded in opponent process theory whereby termination, or offset of an affective process of one valence (either positive or negative), is argued to be associated with the onset of a complimentary affective response of the opposite valence [ 22, 23]. Accordingly, termination of the negative affective state resulting from anticipation of an aversive outcome would, in opponent process terms, be associated with the onset of an opposing positively valenced hedonic response. Consistent with this possibility, Morris (1975) showed that a stimulus that signals successful avoidance, which is presented following the production of an avoidance response, can subsequently be used as a positive reinforcer in its own right [ 24].

In the present study we aimed to address the question of whether successful avoidance of an aversive outcome exhibits the same properties as a reward. Rather than restricting our analysis to behavior, we approached this question by looking directly into the brain by scanning human participants with functional MRI (fMRI) while they performed a simple instrumental conditioning task, in which they could choose to avoid an aversive outcome as well as to obtain a reward. We adopted the rationale that should avoiding an aversive outcome act as a reward, then it should engage similar underlying neural circuitry as that elicited during reward receipt. If, on the contrary, these two processes are found to engage completely distinct and non-overlapping neural circuitry, then this would suggest that avoidance and reward may depend on very distinct neural substrates, providing evidence against a simple reward-based theory of avoidance.

**Figure 1:**
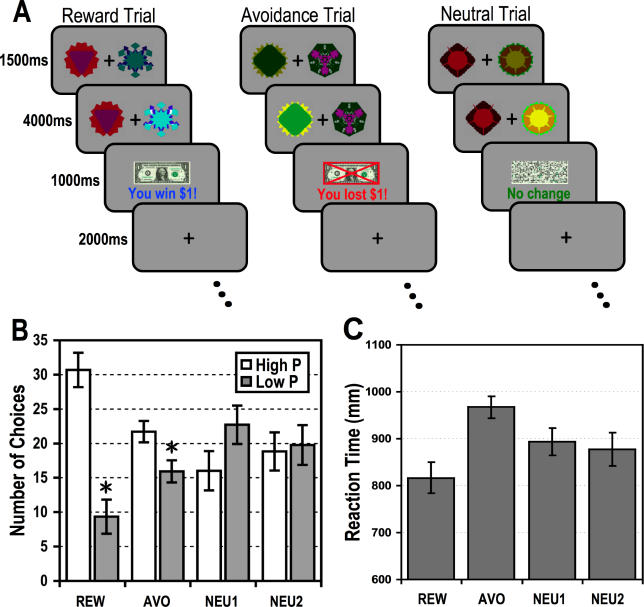
Schematic of Experimental Design (A) In the Reward trials, choice of one action leads to a 60% probability of a reward outcome ($1), and choice of the other action to a 30% probability of reward. In the avoidance condition, participants choose to *avoid* losing money (with 60% versus 30% probability of successful avoidance). Two sets of neutral trials, Neu1 and Neu2, serve as separate baselines for the reward and avoidance conditions, respectively. (B) Behavioral data averaged across all 16 participants showing the total number of responses allocated to the high and low probability actions separately for the reward and avoidance conditions and neutral controls. Participants chose the high probability action significantly more often than the low probability action in both reward and avoidance conditions, but not in the neutral condition (* indicates *p* < 0.05, one-tailed). (C) Plot of reaction times for the different conditions, illustrating a significant difference in reaction times between the reward and avoidance trials.

The instrumental choice task we used was one where participants could win or lose money. There were two main trial types: reward and avoidance. On reward trials participants could choose from one of two actions that led to a high or low probability of obtaining a monetary reward (earning $1), whereas on avoidance trials participants could choose between two actions leading to a high or low probability of avoiding an aversive outcome (losing $1) ( Figure 1A). We used this probabilistic design to enable us to separate out prediction and prediction error signals (see below), as well as to enable us to compare the responses during successful and unsuccessful avoidance.

There is now substantial evidence that the human orbitofrontal cortex (OFC), especially its medial aspect, is involved in coding for the reward value of stimuli in a variety of modalities [ 25– 27]. Of particular note, this region shows robust increases in activity following the receipt of explicit monetary reward outcomes; and moreover, correlates significantly with the magnitude of the outcomes received [ 28– 30]. Thus, the medial OFC is a strong candidate region for encoding the positive reward value of an outcome, particularly that of abstract monetary rewards. On these grounds, we hypothesized that if avoidance of an aversive outcome acts as a reward, then neural activity in the medial OFC should increase following avoidance of an aversive outcome, as well as during receipt of a reward.

In our analysis, in addition to modeling responses at the time of the outcome, we used a computational reinforcement learning model [ 6] to generate signals pertaining to the predicted future reward (or aversive outcome) on each trial from the time the cue stimuli are presented until the time of outcome delivery, as well as for errors in those predictions (discrepancies between expected and actual reward). These signals were then entered into a regression analysis against each participant's fMRI data alongside outcome responses. This allowed us to test for responses to the rewarding and punishing outcomes while at the same time accounting for the effects of reward expectation and prediction error.

## Results

### Behavioral Results

Over the course of the experiment participants showed a statistically significant preference for the action associated with a lesser probability of receiving an aversive outcome: *t*(15) = 1.9, *p* < 0.05, one-tailed; and for the action associated with the greater probability of reward: *t*(15) = 4.34, *p* < 0.01, one-tailed ( Figure 1B), indicating that they had shown avoidance as well as reward conditioning. As expected, participants did not show a statistically significant preference for the actions associated with a greater or lesser probability of neutral feedback in both control conditions: neutral (1): *t*(15) = 1.19, *p* = 0.25, two-tailed; neutral (2): *t*(15) = 0.17, *p* = 0.87, two-tailed. In addition, we ran an analysis of variance to test for a significant interaction effect between probability (High versus Low) and condition (Reward versus Avoidance). We found a significant main effect of probability: *F*(1,15) = 25.14, *p* = 0.00015 and condition: *F*(1,15) = 11.95, *p* = 0.0035; as well as a weak interaction effect between them: *F*(1,15) = 6.43, *p* = 0.022. The number of responses allocated to the high and low probability actions are shown separately for the first and last ten trials of the experiment in Figure S1 to illustrate the effects of learning in both the reward and avoidance trials. Analysis of the reaction time (RT) taken for participants to make a choice in the avoidance and reward conditions revealed that participants had significantly longer RTs for avoidance trials: mean RT = 967.22 ± 93.51 msec: *t*(15) = 3.37, *p* < 0.05, two-tailed, and significantly shorter RTs for reward trials: mean RT = 816.89 ± 130.57 msecs: *t*(15) = 2.63, *p* < 0.05, two-tailed, compared to the neutral trials ( Figure 1C).

### Neuroimaging Results

#### Regions responding to avoidance and reward receipt.

**Figure 2:**
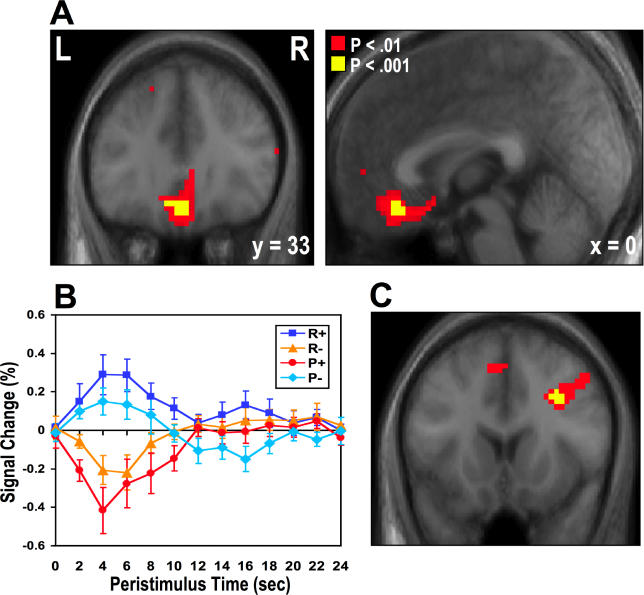
Responses to Outcome in Medial OFC (A) Medial OFC showing a significant increase in activity after avoidance of an aversive outcome as well as after obtaining reward [x = 0, y = 33, z = −18, Z = 3.48, *p* < 0.05] (corrected for small volume using coordinates derived from a previous study) [ 30]. No other brain areas showed significant effects at *p* < 0.001, uncorrected. Voxels significant at *p* < 0.001 are shown in yellow. To illustrate the extent of the activation we also show voxels significant at *p* < 0.01 in red. (B) Time-course plots of peak voxels in the OFC for the four different outcomes: receipt of reward (R+), avoidance of an aversive outcome (P−), missed reward (R−), and receipt of an aversive outcome (P+). The plots are arranged such that time 0 corresponds to the point of outcome delivery. These time courses are shown after adjusting for the effects of expected value and PE (i.e., removing those effects from the data). Non-adjusted time-course data from this region (time-locked to the trial onset and including the effects of expected value) are shown in Figure 3C. (C) Right dorsolateral prefrontal cortex activity was revealed in the opposite contrast to that reported in Figure 2A (i.e., [R _−_ + P _+_] – [R _+_ + P **_−_**]), depicting areas responding more to receiving an aversive outcome and missing reward than to getting reward and avoiding an aversive outcome.

Consistent with our hypothesis, we found that the medial OFC [x = 0, y = 33, z = −18, Z = 3.48], a region previously implicated in responding to receipt of monetary reward, showed increased BOLD responses to the successful avoidance of a monetary loss (P **_−_**) as well as to receipt of monetary reward (R **_+_**) at *p* < 0.001 (uncorrected) ( Figure 2A). No other brain region showed significant effects in this contrast at *p* < 0.001. We extracted trial averaged time-course data from the peak voxel in the medial OFC from each participant and then averaged across participants ( Figure 2B). Activity in this region increases not only to receipt of a rewarding outcome (R _+_), but also following successful avoidance of the aversive outcome (P _−_). Receipt of an aversive outcome leads to a decrease in activity in this region (P _+_). Furthermore, on trials in which a rewarding outcome is omitted, a decrease in activity occurs in the region, mirroring the decrease in activity to receipt of an aversive outcome itself.

**Figure 3:**
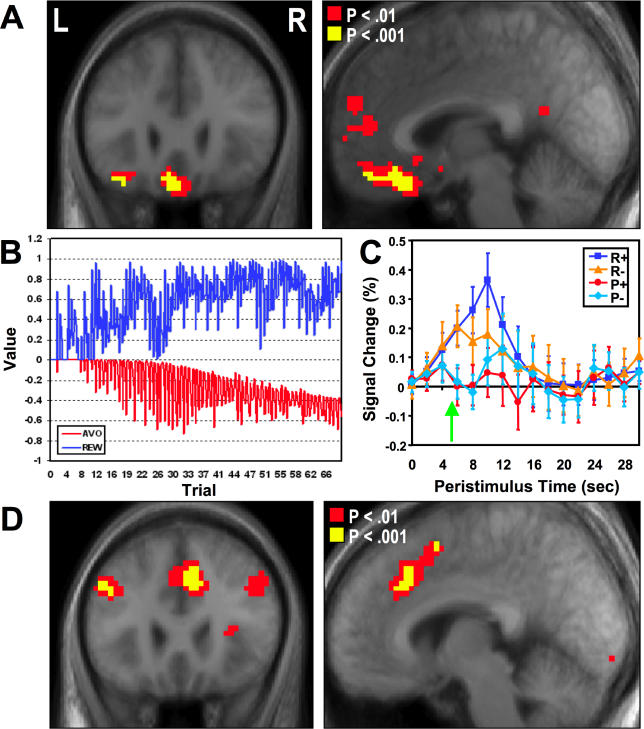
Responses Related to Expected Value (A) Brain areas correlating with expected reward value in both the avoidance and reward trials, revealing significant effects in medial and lateral OFC. (B) Illustration of an expected value signal shown for each trial over the course of the experiment from a typical participant. This signal is generated by the computational model after passing that participant's behavioral data to the model as input. In the reward trials, this value signal increases over time, reflecting the fact that as rewards are obtained over the course of learning, the expected value of the chosen action on the reward trials increases as it is updated over the course of learning. The value signal in the avoidance trial decreases over time, reflecting the fact that as aversive outcomes are obtained over the course of learning, the expected reward value of the currently chosen action decreases as it is updated. (C) Plot of time course taken from the medial OFC showing responses occurring from trial onset. The time of outcome delivery is 4 s into the trial. This plot illustrates an increase in activity from the beginning of the trial on reward trials and a decrease in activity on avoidance trials. The time courses further diverge following the outcome (marked as a green arrow): trials in which a reward is delivered show further increases in activity compared to trials in which a reward is omitted, whereas trials in which an aversive outcome is omitted increase in activity relative to trials where an aversive outcome is delivered (reflecting the effects shown in Figure 2B). (D **)** Brain regions correlating negatively with expected reward value **.** These areas include the bilateral dorsolateral prefrontal cortex and the anterior cingulate cortex.

In order to test for the significance of a direct comparison between the R _+_ and P _−_ conditions and their appropriate neutral baselines, we performed a post-hoc analysis on the time-course data extracted from the medial OFC area. This analysis revealed that both the R _+_ and P _−_ events are associated with significantly increased activity in this region of the medial OFC when compared to their neutral counterparts (R _+_ − N1 _+_: *t* = 3.12, *p* = 0.0044, one-tailed; P _−_− N2 _−_: *t* = 2.27, *p* = 0.021, one-tailed). Moreover, the P _+_− N2 _+_ and R _−_− N1 _−_ events are associated with a significant *decrease* in activity relative to their corresponding neutral events (R _−_: *t* = −2.87, *p* = 0.0071, one-tailed; P _+_: *t* = −2.92, *p* = 0.0065, one-tailed) (See Figure S2). Thus, we provide direct evidence that the medial OFC responds similarly to avoidance of an aversive outcome as it does following receipt of an actual reward. Moreover, neural activity following omission of a reward decreases, just as it does following receipt of an actual aversive outcome (losing money).

To exclude the possibility that these results are critically dependent on the inclusion of prediction error signals as regressors in our fMRI analysis, we performed an additional fMRI analysis whereby we excluded prediction errors from our statistical model. In spite of this, we still observed significant effects in the medial OFC at the time of outcome, indicating that these results are not critically dependent on the presence of prediction error signals in our analysis (see Figure S3).

#### Direct comparison between avoidance of an aversive outcome and reward receipt.

In a direct comparison to test for areas responding more during avoidance of an aversive outcome than during receipt of reward, no brain area showed significant effects at *p* < 0.001. In a contrast to test for regions responding significantly more to receipt of reward than avoidance, activity was found in one region: the medial caudate nucleus [x = 15, y = 18, z = 9, Z = 3.69], at *p* < 0.001.

#### Neural correlates of reward expectation (expected reward value signals).

We also tested for regions responding during expectation of reward. To do this we included as a regressor for each trial type in the neuroimaging analysis, the expected value signal (set at the time of choice) from our reinforcement-learning model. We then tested for areas correlating with expected reward value in the avoidance and reward trials. The expected value signal was found to correlate significantly with activity in the medial [x = −6, y = 30, z = −21, Z = 4.25, *p* < 0.001] and lateral OFC [x = −36, y = 27, z = −21, Z = 4.03, *p* < 0.001] ( Figure 3A), indicating that these regions are involved in coding positive reward expectation (at *p* < 0.001). The area correlating with expected reward value in both trials overlaps with the region of the medial OFC we highlight above as responding to receipt of outcomes. In the reward trial, this value signal increases over time, reflecting the fact that as rewards are obtained over the course of learning, expected reward value increases as it is updated. The value signal in the avoidance trial decreases over time during initial learning, reflecting the fact that as aversive outcomes are obtained, expected reward value decreases as it is updated. Thus, these areas of the medial and lateral OFC show increases in activity as a function of increases in expected future reward value, and decreases in activity as a function of decreases in expected future reward, consistent with expected value signals computed by the computational model (see Figure 3B).

We also tested for regions that correlate negatively with expected reward value, i.e., increasing in activity as expected reward value decreases. This can be thought of as an expected future aversive outcome signal. In the reward trials this signal decreases from trial to trial as learning progresses, whereas in the avoidance trials, this signal increases over time. In both reward and avoidance trials we found evidence for such signals in the left [x = −42, y = 21, z = 33, Z = 4.01, *p* < 0.001] and right dorsolateral prefrontal cortex [x = 48, y = 33, z = 33, Z = 3.34, *p* < 0.001], as well as in the anterior cingulate cortex [x = 6, y = 27, z = 36, Z = 4.35, *p* < 0.001] ( Figure 3D).

#### Prediction error signals.

**Figure 4:**
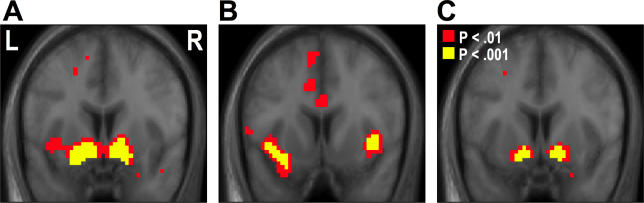
PE-Related Responses (A **)** Ventral striatum (extending from the ventral putamen into the nucleus accumbens proper) correlating with the reward (compared to neutral) PE signal derived from our model. (B) Left and right insula showing significant correlations with an aversive (compared to neutral) PE signal on avoidance trials. (C) Bilateral ventral striatum showing significantly greater PE signals on reward compared to avoidance trials.

We then tested for regions correlating with the reward prediction error (PE) signal derived from our model. Consistent with previous results, we found significant PE activity on the reward trials (when compared to neutral trials) in the ventral striatum (extending from the ventral putamen into the nucleus accumbens proper) [left: x = −15, y = 6, z = −15, Z = 5.52, *p* < 0.001; right: x = 15, y = 6, z = −15, Z = 6.09, *p* < 0.001] ( Figure 4A). Other regions correlating with this PE signal include the medial prefrontal cortex [x = 0, y = 30, z = 18, Z = 5.10, *p* < 0.001] and the left dorsolateral prefrontal cortex [x = −24, y = 30, z = 48, Z = 3.94, *p* < 0.001].

We also tested for PE signals on the avoidance trials. We first looked for areas showing an aversive PE signal, that is, increasing in activity when an aversive outcome was received when unexpected (positive aversive outcome PE), and decreasing activity when an aversive outcome was not received when expected (negative aversive PE). Areas showing significant correlations with this aversive outcome PE signal include the left [x = −39, y = 12, z = −9, Z = 3.62] and right insula [x = 39, y = 15, z = −3, Z = 3.77], posterior ventral thalamus [x = −3, y = −24, z = 0, Z = 5.96], medial prefrontal cortex [x = 0, y = 36, z = 15, Z = 3.71], and posterior midbrain [x = 0, y = −27, z = −27, Z = 4.82, *p* < 0.001] ( Figure 4B). Furthermore, we tested for regions showing a reward PE signal in the avoidance trials; that is, responding with increases in activity on trials where no aversive outcome was received when expected, and decreasing in activity on trials where an aversive outcome was received when unexpected. We did not find any significant correlations with this signal on avoidance trials at *p* < 0.001. Finally, we directly compared PE signals on the reward trials to PE signals on the avoidance trials. This contrast revealed significantly greater PE signals in ventral striatum on reward trials than on avoidance trials [left: x = −12, y = 6, z = −15, z = 4.52, *p* < 0.001; right: x = 15, y = 6, z = −15, z = 4.43, *p* < 0.001] ( Figure 4C).

#### Medial OFC outcome responses—reflecting goal-directed attainment of outcome or prediction error?

**Figure 5:**
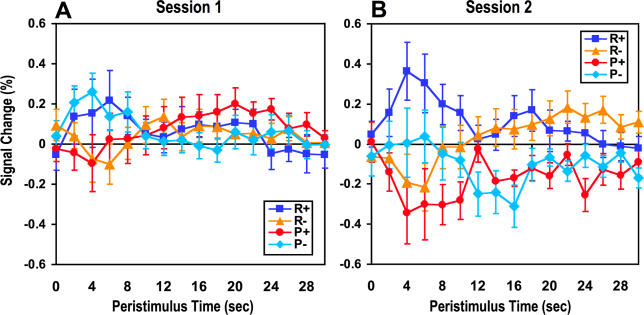
Time-Course Plots in Medial OFC for the Two Sessions Separately Time-course plots showing the trial averaged BOLD signal in the medial OFC, separately for the first (A) and second (B) half of the experiment. The reward response does not decrease toward baseline by the second half of the experiment, nor does the punishment signal increase toward baseline, as would be expected for a PE signal.

Although we fitted PE signals separately in our analysis and thus accounted for the possible confounding effects of PEs (at least as described by our reinforcement learning model), we remained concerned that the medial OFC responses at the time of outcome could reflect some form of residual PE signal not picked up by our PE regressor. As can be seen from the time-course plot in Figure 2B, the signal we observe does appear to resemble a reward PE signal in that there is a positive increase to a not fully predicted omission of an aversive outcome, a positive increase to receipt of a not fully predicted reward, a decrease following omission of reward, and a decrease to receipt of an aversive outcome. In order to exclude a PE account for this medial OFC activity, we plotted the signal in the medial OFC (after adjusting for modeled PE and value signals) separately for the first and second half of the experiment. We reasoned that if the response in the OFC merely reflects a type of residual PE signal then the response to the receipt reward should decrease over time as the reward becomes better predicted. Furthermore, by the same reasoning the signal should also increase over time to punishing outcomes (illustrated in Figure S4). In fact, no such decrease in response to rewarding outcomes or increase in response to punishing outcomes was observed ( Figure 5). Indeed, if anything, activity increases slightly to rewarding and decreases to punishing outcomes by the second phase of the experiment. These results indicate that activity we report in the medial OFC at the time of outcome in the rewarding and avoidance trials is more likely to reflect the hedonic value of successfully attaining a particular goal (which may become stronger over time as the link between actions and their outcomes becomes better learned), rather than constituting a form of PE.

## Discussion

In avoidance learning, an animal or human learns to perform a response in order to avoid an aversive outcome. Here we provide evidence with fMRI that during such learning a part of the human brain previously implicated in responding to reward outcomes, the medial OFC, increases in activity following successful avoidance of the aversive outcome. These results are compatible with the possibility that activity in the medial OFC during avoidance reflects an intrinsic reward signal that serves to reinforce avoidance behavior.

Activity in the medial OFC not only increased after avoiding an aversive outcome or receiving reward, but also decreased after failing to obtain a reward or receiving an aversive outcome. Consequently, this region shows a fully opponent response profile to rewarding and aversive outcomes and their omission [ 22]. This finding suggests the relevance of opponent process theory to avoidance learning, following a recent report of similar underlying processes in pain relief [ 31]. These OFC responses cannot be explained as PE, because activity does not decrease to rewarding outcomes nor increase to aversive outcomes even as these outcomes become better predicted over the course of learning. Rather, responses to rewarding and aversive outcomes in this region likely reflect a positive affective state arising from the successful attainment of reward and a negative affective state from failing to avoid aversive outcome. Similarly, differential activity in this region to avoiding an aversive outcome and missing reward may reflect a positive affective response to successfully avoiding an aversive outcome and a negative affective state arising from failure to obtain a reward. Thus, our findings indicate that medial OFC activity at the time of outcome reflects the affective (or reinforcing) properties of goal attainment. This is bolstered by a number of previous neuroimaging studies that implicate this region in responding to receipt of many different types of reward including money, but also attractive faces, positively valenced face expressions, pleasant music, pleasant odors, and foods [ 27– 30, 32, 33]. While other studies have reported a role for the medial OFC in complex emotions such as “regret,” which may contain both positive and negative affective components [ 34], the results of these previous reward studies, when combined with the present findings, suggest a specific (though not necessarily exclusive) role for the medial OFC in encoding the positive hedonic consequences of attaining both extrinsic and intrinsic reward. The finding described here, of a specific role for the medial OFC in signaling goal-attainment, adds to burgeoning literature implicating the ventromedial prefrontal cortex as a whole, in goal-directed decision making and motivational control [ 35– 42].

Alongside outcome-related responses, activity in the medial OFC was also found to correlate with an expected reward value signal derived from our reinforcement-learning model. This is reflected by an increase in activity following the onset of reward trials, during which (following learning) delivery of reward is expected, as well as by a decrease in activity following the onset of avoidance trials, during which (following learning) delivery of an aversive outcome is expected. This expected reward value signal co-exists in the same region of the medial OFC found to respond to reward outcomes. While we observe the same region of the medial OFC responding during both anticipation and receipt of reward, limits in the spatial resolution of fMRI preclude us from determining whether the same population of neurons within the medial OFC are sensitive to both reward expectation and receipt of reward outcomes, or if two distinct but spatially intermingled populations of neurons within this region exhibit selective responses to either expectation or receipt of reward. Nevertheless, we also found a region of the lateral OFC responding during reward expectation that did not respond during receipt of reward, indicating that these two components of reward processing are at least partially dissociable [ 43].

The findings reported here also help to address previous discrepancies in the reward neuroimaging literature as to the differential role of the medial versus lateral OFC in processing rewarding and aversive outcomes [ 29, 44, 45]. In the present study we show that the medial OFC responds to reward outcomes (as well as following successful avoidance of aversive outcomes), whereas both medial and lateral OFC responds during anticipation of reward. Indeed, when we tested for regions showing increases in activity to receipt of aversive outcome or omission of reward, we found a region of the lateral prefrontal cortex extending down to the lateral orbital surface with this response profile, implicating this region in responding to monetary an aversive outcomes [ 30]. These findings suggest the possibility that dissociable activity within the medial versus lateral OFC may be evident during receipt of rewarding and punishing events, but not during their anticipation.

We also tested for regions of the human brain involved in encoding PE signals during both reward and avoidance learning. We found a fully signed reward PE signal in the ventral striatum on reward trials, whereby activity increases following unexpected delivery of reward, but decreases following unexpected omission of reward (as shown previously [ 10, 46]). However, we did not find an aversion-related PE signal in the ventral striatum on avoidance trials, whereby signals increase following expected delivery of an aversive event but also decrease following unexpected omission of the aversive event. This is in direct contradiction of previous studies that have reported such signals during aversive learning with pain or even a least preferred food stimulus [ 31, 47– 49]. In our study, PE signals were significantly greater in reward trials than avoidance trials in this region, even following presentation of an unexpected aversive stimulus. Yet, decreases (rather than increases) in activity in the ventral striatum during aversive learning have been reported in at least a few other studies, specifically those featuring receipt of monetary aversive outcomes [ 50, 51]. One plausible explanation for these apparent contradictory findings is that monetary loss as a secondary reinforcer may be processed differently in the ventral striatum than more primary punishing stimuli such as aversive flavors or pain.

The main finding of this study is that the medial OFC responds during successful avoidance of aversive outcome as well as during receipt of explicit rewards. An important caveat is that the results presented here do not necessarily provide a complete explanation for why, in some animal learning studies, behavior is maintained even after complete avoidance of such outcomes [ 15]. Unlike in those studies, avoidance behavior in the present study could have been maintained by virtue of the fact that the participants continued to receive aversive outcomes from time to time. Nonetheless, it is certainly plausible that similar opponent reinforcement mechanisms to those shown here could also play a role even when a punisher can be completely avoided. However, in this case, additional mechanisms may also come into play in order to account for resistance to extinction, such as the onset of habitual control processes (see Mackintosh, 1983, Chapter 6) [ 52].

A role for the medial OFC in responding following avoidance of an aversive outcome provides an important insight into the conundrum of avoidance learning. It seems that the same neural circuitry is recruited during avoidance of an aversive outcome as is recruited during receipt of reward. Consequently, this neural avoidance signal may itself act as a reinforcer, and just as a reward does, bias action selection so that actions leading to this outcome are chosen more often. More generally, our results point to a key role for the medial OFC in mediating the affective components of goal attainment, whether the goal is to obtain reward or avoid an aversive outcome.

## Materials and Methods

### Participants.

Participating in the experiment were16 right-handed healthy normal individuals (seven females, nine males; mean age: 25.81 ± 7.87; range: 19–48). The participants were pre-assessed to exclude those with a prior history of neurological or psychiatric illness. All participants gave informed consent and the study was approved by the Institutional Review Board of the California Institute of Technology.

### Stimuli and task.

Each trial in the monetary instrumental task began with the simultaneous presentation of one of two pairs of fractal stimuli. Each pair signified the onset of one of four trial types: Reward, Avoidance, Neutral (1), and Neutral (2), whose occurrence was fully randomized throughout the experiment (see Figure 1A). The specific assignment of fractal pairs to a given trial type was fully counterbalanced across participants. The participant's task on each trial was to choose one of the two stimuli by selecting the fractal to the left or right of the fixation cross via a button box (using their right hand). Once a fractal had been selected, it increased in brightness and was followed 4 s later by visual feedback indicating either a reward (a picture of a dollar bill with text below saying “You win $1”), an aversive outcome (a red cross overlying a picture of a dollar bill with text below saying “You lost $1”), neutral feedback (a scrambled picture of a dollar bill with text below saying “No change”), or nothing (a blank screen with a cross hair in the center).

In the reward trials, when participants chose the high probability action, they received monetary reward with a 60% probability; on the other 40% of trials they received no feedback. Following choice of the low probability action, they received monetary reward on only 30% of trials; otherwise, they obtained no feedback (on the remaining 70% of trials). Similarly on the avoidance trials, if participants chose the high probability action they received no feedback on 60% of trials, on the other 40% they received a monetary loss, whereas choice of the low probability action led to no outcome on only 30% of trials, while the other 70% were associated with receipt of the aversive outcome. We also included two affectively neutral conditions, Neu1 and Neu2, as baselines for the Reward and Avoidance conditions, respectively. On Neu1 trials, participants had a 60% or 30% probability of obtaining neutral feedback (a scrambled dollar); otherwise, they received no feedback. On Neu2 trials participants had a 60% or 30% probability of receiving no feedback; otherwise, they received neutral feedback. These neutral conditions allowed us to control for motor responses as well as for simple visual effects relating to the presentation or absence of feedback. Participants underwent two ∼ 20 min scanning sessions (two sessions), each consisting of 160 trials (40 trials per condition) for session. All four conditions were pseudorandomly intermixed throughout the two sessions.

Prior to the experiment, participants were instructed that they would be presented with four pairs of fractals, and on each trial they had to select one of these. Depending on their choices they would win money, lose money, obtain a neutral outcome (scrambled dollar which means no change in income), or receive no feedback. They were not told which fractal pair was associated with a particular outcome. Participants were instructed to try to win as much money as possible. Participants started the task with an endowment of $35 and were told that any losses they incurred would be subtracted from this total, whereas any gains they incurred would be added to the total. As per instructions, participants were paid according to their performance at the end of experiment, receiving on average of $40.33.

### Imaging procedures.

The functional imaging was conducted by using a 3 Tesla Siemens TRIO MRI scanner to acquire gradient echo T2* weighted echo-planar images (EPI) images with BOLD (Blood Oxygenation Level Dependent) contrast. We used a tilted acquisition sequence at 30 ° to the AC-PC line to recover signal loss from dropout in the medial OFC [ 53]. In addition, we used an 8-channel phased array coil which yields a ∼ 40% signal increase in signal in the medial OFC over a standard head coil. Each volume comprised 32 axial slices of 3-mm thickness and 3-mm in-plane resolution, which was acquired with a TR of 2 s. A T1-weighted structural image was also acquired for each participant.

### Advantage learning model.

The advantage learning model used here is identical to that used by O'Doherty et al. [ 54]. Advantage learning [ 55] uses a temporal difference (TD) learning rule to learn value predictions of future reward [ 5]. In temporal difference learning, the prediction 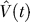 of the value *V*( *t*) at any time *t* within a trial is calculated as a linear product of the weights *w _i_* and the presence or absence of a conditioned stimulus (CS) at time *t,* coded in the stimulus representation vector *x _i_*( *t*):



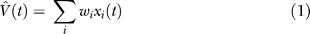



Learning occurs by updating the predicted value of each time-point *t* in the trial by comparing the value at time *t* + 1 to that at time *t,* leading to a PE or



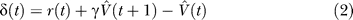



where *r*( *t*) is the reward at time *t.*

The parameter *γ* is a discount factor that determines the extent to which rewards that arrive earlier are more important than rewards that arrive later on. In the present study we set *γ* = 1. The weights *w _i_* are then updated on a trial-by-trial basis according to the correlation between PE and the stimulus representation:



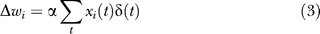



where *α* is the learning rate.

We assigned six time points to each trial, and used each participant's individual event history as input. We set *r*( *t*) to 1, 0, or −1 to denote receipt of a reward outcome, no outcome, or an aversive outcome, respectively. On each trial, the CS was delivered at time point 1, the choice was made at time point 2, and the reward was delivered at time point 6. For the analysis, reward PEs are calculated for the specific CS that was illuminated (i.e., at the time of choice, where 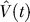
was generated based on just one of the two stimuli shown).

The PE signal used in the fMRI analysis is a variant of *δ*( *t*) known as the advantage PE signal *δ ^A^*( *t*):



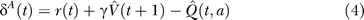



where *Q*( *t*, *a*) corresponds to the value of the specific chosen action a at time *t,* and *V*( *t*) is the value of the state at the current time *t* as calculated in Equation 1 above. These action values are used to determine the probability of choosing a given action using a logistic sigmoid:



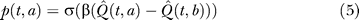



where *β* is an inverse temperature that determines the ferocity of the competition. This probability is then used to define the value of the initial state at *t* = 1 as:



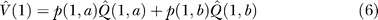



In order to find optimal model parameters, we calculated the log likelihood fit of the actual choices made by participants according to advantage learning, for a variety of learning rates *(α)* and inverse temperature parameters *(β).* These optimal parameters, which were obtained separately for different trial types: *α* = .68 and *β* = .89 for reward, *α* = .12 and *β* = .64 for avoidance, *α* = .19 and *β* = .75 for neutral (1), and *α* = .2 and *β* = .82 for neutral (2) were used to generate the actual regressors for the fMRI data analysis.

### Imaging data analysis.

Image analysis was performed using SPM2 (Wellcome Department of Imaging Neuroscience, Institute of Neurology, London, United Kingdom). To correct for participant motion, the images were realigned to the first volume, spatially normalized to a standard T2* template with a re-sampled voxel size of 3 mm ^3^, and spatial smoothing was applied using a Gaussian kernel with a full-width at half-maximum (FWHM) of 8 mm. Time series describing expected reward values and PEs were generated for each participant for each trial in the experiment by entering the participant's trial history into the advantage RL model. These sequences were convolved with a hemodynamic response function and entered into a regression analysis against the fMRI data. We have modeled value responses (reward expectation) as a tonic signal beginning at the time of presentation of the CS and continuing until receipt of the reward. Thus, we use a full-value signal as implemented in temporal difference learning models. In addition to value and PE, separate regressors were created for different outcomes to model activity at the time of the outcome: rewarded reward trial (R **_+_**), unrewarded reward trial (R **_−_**), punished avoidance trial (P **_+_**), non-punished avoidance trial (P **_−_**), etc. These outcome regressors were orthogonalized with respect to PE, so that the PE regressor was ascribed all of the variance common between the outcome responses and PE. Consequently, any activity loading on the outcome regressors corresponds to that portion of the variance explained by the response to the outcome itself and not PE. In addition, the six scan-to-scan motion parameters produced during realignment were included to account for residual effects of movement.

Linear contrasts of regressor coefficients were computed at the individual participant level to enable comparison between the Reward, Avoidance, and Neutral trials. The results from each participant were taken to a random effects level by including the contrast images from each single participant into a one-way analysis of variance with no mean term. The specific contrast used to generate the results in Figure 2 is: [R _+_ + P _−_] – [R _−_ + P _+_] i.e., a test of those areas showing greater responses to obtaining reward and avoiding aversive outcome compared to obtaining aversive outcome and missing reward. The simple contrast of [P **_−_** − P _+_] also revealed significant effects in the same region (at *p* < 0.001, uncorrected) as did [R _+_ − R _−_] ( *p* < 0.005, uncorrected).

The structural T1 images were co-registered to the mean functional EPI images for each participant and normalized using the parameters derived from the EPI images. Anatomical localization was carried out by overlaying the *t*-maps on a normalized structural image averaged across participants and with reference to an anatomical atlas.

For the time-course plots, we located functional ROIs within individual participant's medial OFC and extracted event- related responses from the peak voxel for that participant using SPM2. Data from three out of 16 participants were not included in the time-course plots, as effects were not observed in the medial OFC for those participants at the minimum threshold of *p* < 0.05 (uncorrected). Event-related responses for the reward and avoidance trials are plotted with respect to their appropriate neutral baselines.

## Supporting Information

Figure S1 Effects of Learning Plot of the choices allocated to the high and low probability actions for the first and last ten trials of the experiment, to illustrate the effects of learning. Data for reward and avoidance trials are shown separately.
(846 KB TIF)

Figure S2 Medial OFC Responses to Outcomes Compared to Neutral Baselines Plot of the mean signal change in the medial OFC following receipt of each of the possible outcomes [R+, P−, P+, R−] when compared to its corresponding neutral baseline. The evoked BOLD response following each outcome is significantly different from its corresponding neutral baseline at *p* < 0.05 or lower.
(1.3 KB TIF)

Figure S3 Significant Effects in Medial OFC to Outcomes without including Prediction Error Signals in FMRI Analysis Results from an identical contrast to that shown in Figure 2 (i.e., [R _+_ + P _−_] – [R _−_ + P _+_]) derived from an fMRI analysis in which prediction error signals are excluded from the statistical model. Significant effects in the medial OFC (x = 3, y = 27, z = −24, Z = 3.30) at the time of outcome are still found in this analysis at *p* < 0.001, which rules out the possibility that the effects reported in Figure 2 are critically dependent on the inclusion of prediction error signals in the fMRI analysis. Furthermore, the simple effects of R+ and P− compared to their neutral baselines (as shown in Figure S2 for the full model) remain significant at the *p* < 0.05 level.
(3.8 KB TIF)

Figure S4 Separate Plots of Model-Estimated Prediction Errors for the Two Experimental Sessions Model-estimated prediction errors for each of the different possible outcomes (at the time of outcome) are shown separately for session 1 and session 2. This reveals that there is in fact a marked decrease in prediction error signals from session 1 to session 2, following receipt of a rewarding outcome and an increase in such signals following receipt of an aversive outcome on avoidance trials. This response profile is inconsistent with what we actually observe in the medial OFC, thereby lending support to our claim that responses in the medial OFC at the time of outcome are unlikely to reflect prediction error signals.
(864 KB TIF)

Figure S5 Subjective Pleasantness Ratings for the Fractal Stimuli The plot shows the change in pleasantness ratings for the fractal stimuli associated with each of the different outcomes from before to after the experiment. Both stimuli present in the avoidance trials showed a significant decrease in their pleasantness from before to after conditioning: Avo Hi: *t*(15) = 2.81, *p* < 0.05, one-tailed; Avo Lo: *t*(15) = 1.88, *p* < 0.05, one-tailed.
(22 KB GIF)

## Abbreviations

fMRI: functional MRI
OFC: orbitofrontal cortex
PE: prediction error
RT: reaction time
